# Activation of Ras Requires the ERM-Dependent Link of Actin to the Plasma Membrane

**DOI:** 10.1371/journal.pone.0027511

**Published:** 2011-11-21

**Authors:** Tobias Sperka, Katja J. Geißler, Ulrike Merkel, Ingmar Scholl, Ignacio Rubio, Peter Herrlich, Helen L. Morrison

**Affiliations:** 1 Morrison Laboratory, Leibniz Institute for Age Research – Fritz Lipmann Institute (FLI), Jena, Germany; 2 Herrlich Laboratory, Leibniz Institute for Age Research – Fritz Lipmann Institute (FLI), Jena, Germany; 3 Institute of Molecular Cell Biology, Centre for Molecular Biomedicine, Friedrich-Schiller-University, Jena, Germany; Alexander Flemming Biomedical Sciences Research Center, Greece

## Abstract

**Background:**

Receptor tyrosine kinases (RTKs) participate in a multitude of signaling pathways, some of them via the small G-protein Ras. An important component in the activation of Ras is Son of sevenless (SOS), which catalyzes the nucleotide exchange on Ras.

**Principal Findings:**

We can now demonstrate that the activation of Ras requires, in addition, the essential participation of ezrin, radixin and/or moesin (ERM), a family of actin-binding proteins, and of actin. Disrupting either the interaction of the ERM proteins with co-receptors, down-regulation of ERM proteins by siRNA, expression of dominant-negative mutants of the ERM proteins or disruption of F-actin, abolishes growth factor-induced Ras activation. Ezrin/actin catalyzes the formation of a multiprotein complex consisting of RTK, co-receptor, Grb2, SOS and Ras. We also identify binding sites for both Ras and SOS on ezrin; mutations of these binding sites destroy the interactions and inhibit Ras activation. Finally, we show that the formation of the ezrin-dependent complex is necessary to enhance the catalytic activity of SOS and thereby Ras activation.

**Conclusions:**

Taking these findings together, we propose that the ERM proteins are novel scaffolds at the level of SOS activity control, which is relevant for both normal Ras function and dysfunction known to occur in several human cancers.

## Introduction

The small G-protein Ras functions as a molecular switch relaying extracellular stimuli to diverse intracellular effector pathways, which are responsible for controlling proliferation, motility and differentiation. Because of this central role Ras activity and its downstream signaling pathways must be tightly regulated. At the level of Ras the major determinants currently known are guanine nucleotide exchange factors (GEFs), which catalyze the loading of Ras with GTP replacing tightly bound GDP, and GTPase-activating proteins (GAP), which down-regulate the activity state by enhancing Ras-bound GTP hydrolysis. Specificity of GEF activity e.g. Son of sevenless (SOS) is linked not only to active RTKs through the adaptor protein, Growth factor receptor-bound protein 2 (Grb2), but is also influenced in its activity through interaction with membrane lipids [1], [2], [3]. Further but less well understood complexity of the Ras pathway has been created by the identification in the plasma membrane of nanoclusters of proteins and lipids which are thought to concentrate the components of effector cascades [4]. Also by the finding of scaffold proteins (e.g. kinase suppressor of Ras, KSR, and sprouty-related proteins, (spred) thought to coordinate kinetics of the downstream signaling components and preventing activation of physiologically inappropriate signals [5], [6].

We discovered previously an additional level of regulation of the Ras dependent MAP kinase pathway: Co-receptors specific for a given RTK focus the MAP kinase activation to this receptor [7], [8]. Our observations triggered our interest in defining at what level this control was exerted. Most RTKs require co-receptors such as integrins or other cellular adhesion molecules [7], [8], [9]. On the extracellular side, one of the functions of co-receptors appears to be the local enrichment or proper presentation of receptor ligands [10], [11]. On the intracellular side, the cytoplasmic domains of co-receptors are required for RTK-dependent signaling [12], [13], [14]. Moreover, we identified a new component required for MAP kinase activation – the filamentous actin (F-actin)-binding protein ezrin (or other members of the ezrin-radixin-moesin (ERM) family) that connects the actin cytoskeleton with the plasma membrane. Initial evidence suggests that the ERM proteins bind to the cytoplasmic domain of the co-receptor and from this location required for growth factor induced Ras-MAP kinase activation. However, the precise mechanism of their action has remained elusive to date.

In the present study, we explore how the ERM proteins precisely affect the MAP kinase pathway. We localize the step catalyzed by the ERM proteins to the activation of Ras. Growth factor induced Ras activation is severely inhibited or even abolished by either the disruption of the interaction of the ERM proteins with co-receptors, by siRNA dependent down-regulation of ERM proteins, by the expression of dominant-negative mutants of ezrin, or by the disruption of F-actin. The actin-associated ezrin carries independent binding sites for the co-receptor, for Ras and for the autoinhibitory Dbl homology (DH) domain of the GEF Son of sevenless (SOS), stabilizing a complex consisting of receptor, co-receptor, Grb2, SOS, ezrin-actin and Ras. Mutations of these binding sites destroy the interactions and inhibit the activation of Ras. Furthermore, ezrin (used as an ERM protein prototype) not only serves as a scaffold assembling the partners Ras and SOS, but also appears to be an important regulator of SOS activity. Thus, our data reveal a novel aspect of Ras signaling that may be relevant to normal physiology as well as human cancer.

## Results

### Ezrin domains required for the catalysis of signal transduction

We established the requirement of the ERM proteins for Ras signaling by three independent methods and in several cell lines. First, we overexpressed a soluble peptide – the cytoplasmic tail of the co-receptor CD44 – that sequesters the ERM proteins [10], [15], [16]. As a measure of Ras activity, we used Erk phosphorylation. The wild type peptide, but not a mutant defective in ERM protein binding (Figure S1A), strongly reduced the basal and platelet-derived growth factor (PDGF)-induced phosphorylation of Erk (Figure S1B). Kinetic resolution of PDGF signaling revealed attenuated Erk activation characterized by a shifted phosphorylation maximum (from 1 to 5 minutes) and lower fold-induction (from 19 to 5 times; Figure S1C, D). Thus, removal of the ERM proteins from the co-receptor interferes with signaling.

**Figure 1:**
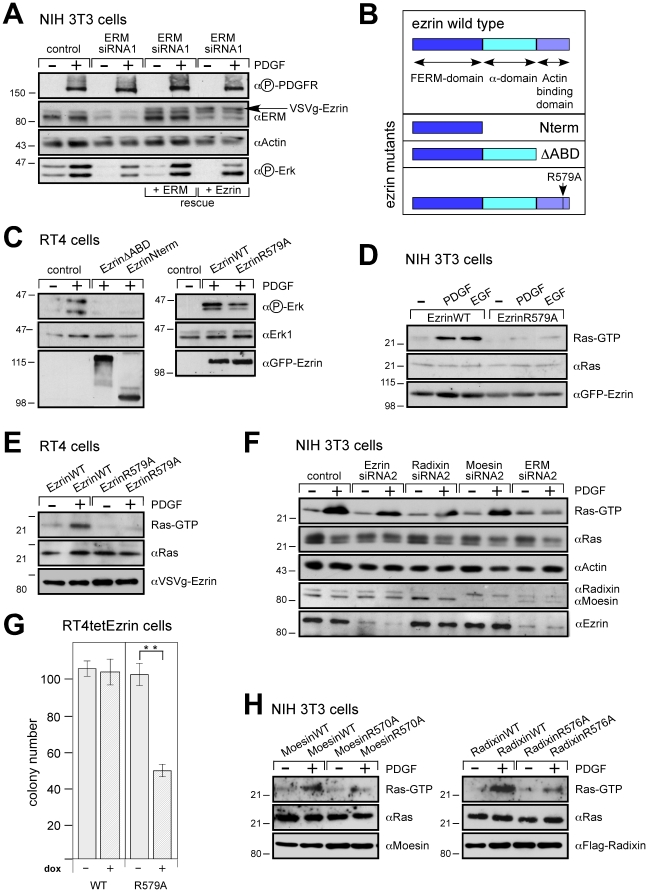
ERM proteins are necessary for PDGFR signaling. **A**, Down-regulation of ERM proteins reduces PDGF-dependent Erk phosphorylation. NIH 3T3 cells plated at low density were treated with a combination of siRNA SMARTpools against mouse ERM proteins for 24 hours. For exogenous reconstitution of human ERMs, cells were transfected with plasmid DNA coding for human ezrin-VSVg, radixin-Flag and untagged moesin or ezrin-VSVg alone. Cells were serum starved overnight prior to treatment with PDGF for 5 min. Lysates were immunoblotted as indicated. **B**, Schematic representation of the architecture of ezrin mutants. **C**, Ezrin mutants inhibit PDGF-dependent Erk phosphorylation. RT4 cells at low density were transfected with either empty vector (control) or ezrin mutants (ezrinNterm-GFP or ezrin deleted in the Actin-Binding-Domain ezrinΔABD-GFP) (left panel) ezrin wild type or ezrin mutant R579A (right panel). Cells with high GFP expression were sorted by FACS, replated at low cell density and serum starved overnight prior to induction with PDGF for 5 min. Lysates were immunoblotted as indicated. **D**, NIH 3T3 cells were plated at a low density, co-transfected in a 5∶1 ratio with constructs encoding either ezrin wild type-GFP or ezrin mutant-GFP and with a hygromycin resistance construct, selected by hygromycin for 1 day, and serum starved overnight prior to treatment with PDGF or EGF for 3 min. For Ras-GTP levels, lysates were treated with GST-Raf1-RBD (Ras-binding domain, RBD). Co-precipitated proteins were immunoblotted with antibodies against Ras. Lysates were immunoblotted as indicated. **E**, Ezrin R579A also inhibits PDGF-dependent Ras activation in RT4 cells. Experiment as in D, except ezrin constructs encoding either ezrin wild type-VSVg or ezrin mutant-VSVg were used. **F**, Down-regulation of individual ERM proteins using a cocktail of specific siRNAs reduces PDGF-dependent Ras activation in NIH 3T3 cells. Ras activity was determined as in **D**. **G**, Ezrin mutant, but not wild type ezrin, inhibits agar colony formation in RT4 cells. Dox-inducible ezrin wild type- or mutant-expressing cells were generated and placed in soft agar (− and + dox). Results represent mean absolute colony number ± s.d. of at least three independent experiments, **P<0.01 using student's t-test. **H**, The F-actin-binding mutants of moesin (R570A, middle panel) and radixin (R576A, right panel) inhibit PDGF-dependent Ras activation in NIH 3T3 cells. Experiment set up as in D. In all panels lysates immunoblotted as indicated. The results are representative of at least three independent assays and each panel represents experiments from the same blot and the same exposure.

Second, down-regulation of the ERM proteins by two independent siRNA cocktails directed against all three ERM proteins lowered PDGF-induced Erk phosphorylation, but did not impair the activation of the PDGF receptor (PDGFR) itself (Figure 1A; Figure S1E, F). As will be shown below, each of the three ERM proteins contribute to activation of signaling, at least in NIH3T3 cells (Figure 1F). Importantly, overexpression of all three human ERM proteins or of ezrin alone rescued the mouse-siRNA-dependent inhibition (Figure 1A). We conclude that the ERM proteins are essential for PDGF signaling towards Erk.

Third, we interfered with ERM protein functions by mutations in ezrin. Thereby, we also defined ezrin domains involved in Ras activation. Ezrin mutants were overexpressed to compete with endogenous ERM proteins for cellular interactions. We first constructed ezrin with deletions in the C-terminus (Figure 1B; Nterm and ΔABD deleting the actin-binding domain). These mutant proteins are still able to bind to co-receptors at the plasma membrane, but cannot interact with F-actin. Stable overexpression of these deletion mutants (as GFP fusions) abolished PDGF-induced Erk phosphorylation (Figure 1C, left panel). Because the deletions might abolish more than just the binding of ezrin to actin, we used ezrin R579A (see Figure 1B), a mutant that is completely defective in F-actin binding, but is otherwise functionally intact [14]. Overexpression of this mutant, but not of wild type ezrin, reduced PDGF-dependent and IL-6-dependent Erk phosphorylation (Figure 1C, right panel; Figure S2A, B). Of note, ezrin R579A was specific for Ras-dependent signaling in as much as it did not interfere with PKC-induced Erk phosphorylation (Figure S2B) nor with IL-6-dependent STAT3 activation, which is independent of Ras (Figure S2B). In conclusion, F-actin binding or an unknown step linked to the ezrin/F-actin interaction is crucial for PDGF signaling to Erk, possibly at the level of or upstream of Ras.

### Ezrin R579A prevents Ras activation

To address whether the mutants directly interfere with the activation of Ras, we measured the PDGF-dependent loading of GTP onto Ras. Overexpression of ezrin R579A (Figure 1C,D) inhibited the activation of Ras. As a consequence of reduced Ras activation, doxycycline-induced expression of ezrin R579A inhibited agar colony formation of RT4 cells, which are driven by mutated ErbB2 (Figure 1G). Interestingly, the effect of ezrin was not limited to the signaling by the PDGFR, but was important for other RTKs too. Epithelial growth factor (EGF)-induced Ras activation was also abolished (Figure 1D), suggesting a general requirement for the ERM proteins in RTK-induced Ras activation. In addition, the contribution of the individual ERM proteins to signal transduction was examined using the corresponding mutants radixin R576A and moesin R570A. Overexpression of each mutant in NIH 3T3 cells inhibited spontaneous and PDGF-induced Ras activation (Figure 1H). Specific siRNA down-regulations (Figure 1F) showed that single knockdowns had partial inhibitory effects on Ras activation, with the combination of all three siRNAs being the most effective. Thus, all three ERM proteins participate in catalyzing the signal transduction to Ras and the overexpression of any one of the mutants competes with the functions of all three wild type proteins.

**Figure 2:**
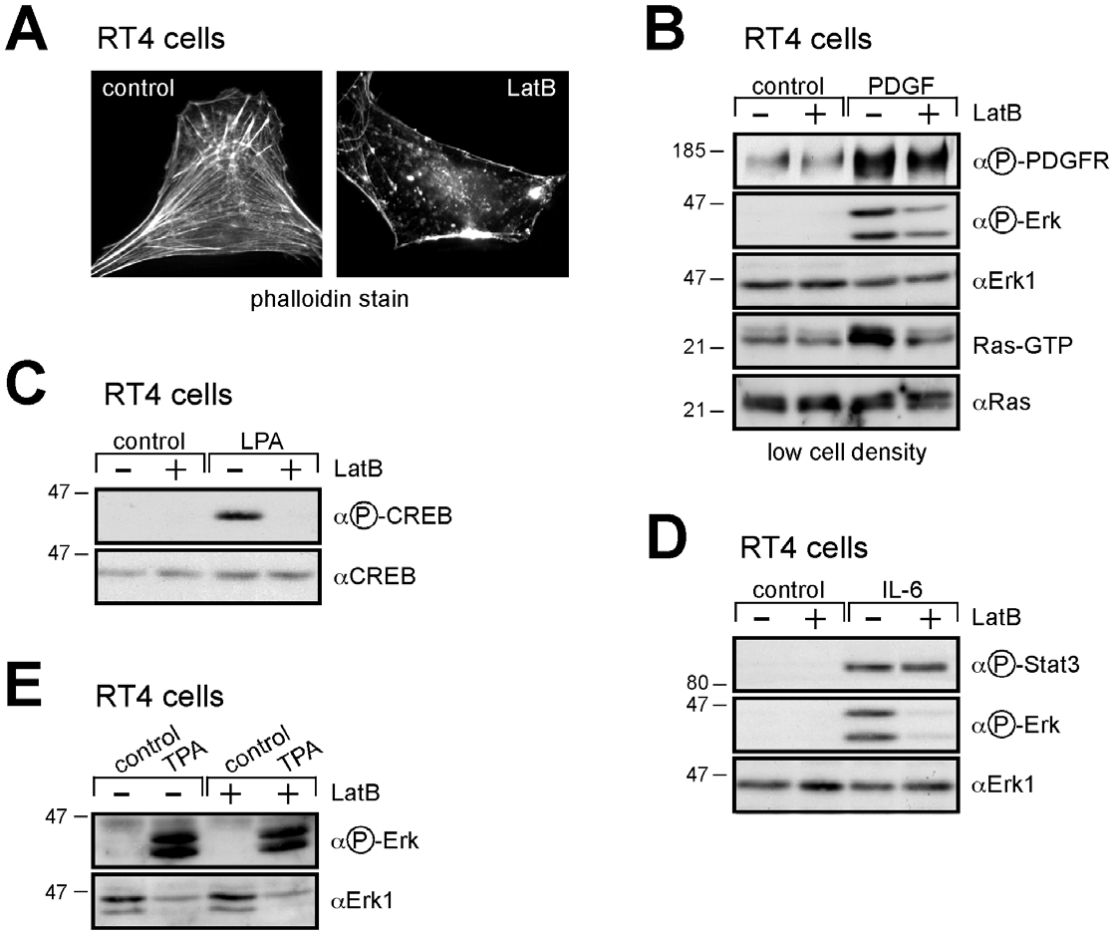
Latrunculin B mimics the ezrin mutants. **A** Reduction in actin filaments by treatment with latrunculin B. The parental schwannoma cells RT4 were plated at low density and treated with latrunculin B (1.25 µM, 10 min). Cells were processed as described in material and methods (scale bar 10 µm). **B** Latrunculin B inhibits signaling. RT4 cells at low density were serum starved overnight, then treated with latrunculin B (1.25 µM, 5 min) prior to treatment with PDGF (10 ng/ml, 5 min). Lysates were treated with GST-Raf1-RBD (to pulldown Ras-GTP). Co-precipitated proteins were immunoblotted with antibodies against Ras. Lysates immunoblotted as indicated. **C**, **D**, **E** Specific interference with signaling by latrunculin B. RT4 cells prepared and treated with latrunculin B as in panel **A**, then stimulated with LPA (20 µM, 5 min, panel **B**), IL-6 (1 ng/ml, 5 min, panel **C**) or TPA (100 ng/ml, 5 min, panel **D**). Lysates immunoblotted as indicated. The results are representative of at least three independent assays and each panel represents experiments from the same blot and the same exposure.

Because ezrin R579A is defective in binding to F-actin, we investigated whether actin itself has a role in Ras activation. Latrunculin B, an inhibitor of actin polymerization (Spector et al, 1983), caused a reduction of stress fibers within minutes (Figure 2A). It also inhibited PDGF-induced Ras activation and downstream signaling to Erk (Figure 2B), while phosphorylation of the receptor (Figure 2B) and its association with Grb2 (not shown) were not affected. Interestingly, its effect on signaling was not indiscriminate. While latrunculin B specifically interfered, in addition to PDGF signaling, with the lysophosphatidic acid-induced phosphorylation of CREB (Figure 2C) and of Erk (not shown), and the IL-6-induced phosphorylation of Erk (Figure 2D), it did not inhibit the activation of STAT3 in response to IL-6 (Figure 2D). Additionally, PKC-dependent Erk phosphorylation in response to phorbol ester, which bypasses Ras, was not affected (Figure 2E). Thus, despite the general disruption of the actin cytoskeleton caused by this inhibitor (which is likely to be lethal over a longer observation period), it exerts, at least at this early time following growth factor stimulation, a very specific effect on signal transduction through the Ras-Erk pathway.

### Ezrin engages SOS and Ras

**Figure 3:**
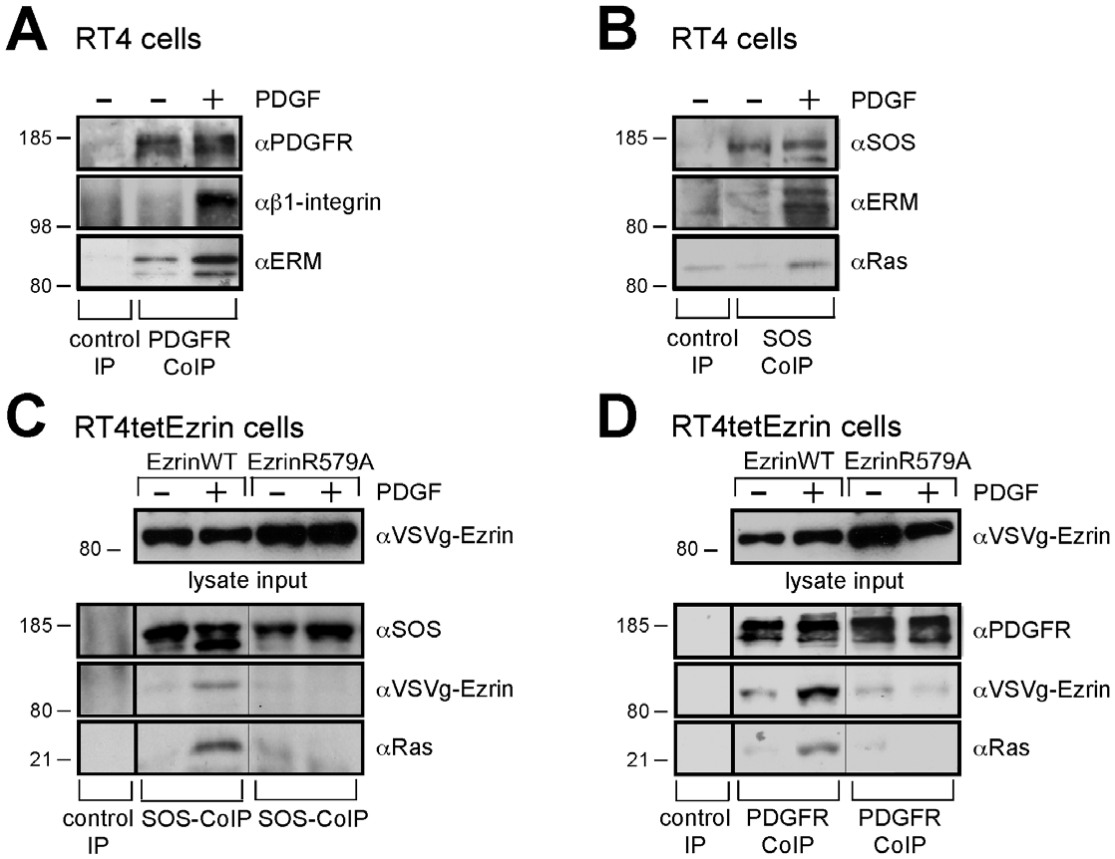
Ezrin engages in a complex with SOS and Ras. **A**, Endogenous ERM proteins associate with the PDGFR and its co-receptor β1-integrin. RT4 cells were plated at low density, serum starved overnight and treated with PDGF for 1 min. Proteins co-immunoprecipitated (CoIP) with PDGFR were immunoblotted, rabbit lgG was used for control IP. **B**, Endogenous ERM proteins associate with SOS and Ras. RT4 cells treated as in **A**. SOS was immunoprecipitated by a rabbit SOS-specific antibody, rabbit lgG was used for control IP. Coimmunoprecipitated proteins were immunoblotted. **C**, Ezrin R579A cannot associate with SOS and Ras. RT4 cells expressing dox-inducible ezrin wild type or R579A mutant were plated at low density, serum starved overnight and incubated with dox prior to treatment PDGF for 1 min. Proteins co-immunoprecipitated with SOS (as in **B**) were immunoblotted as indicated. **D**, Ezrin R579A cannot associate with the PDGFR and Ras. Cells were treated as in **C** followed by PDGFR-CoIP (as in **A**). Proteins co-immunoprecipitated with PDGFR were immunoblotted as indicated. The results are representative of at least three independent assays and each panel represents experiments from the same blot and the same exposure.

Because the actin-binding function of ezrin is required for Ras activation, we speculated on the existence of an actin-associated protein complex involving ezrin. Such a multiprotein complex was indeed confirmed and its composition defined by immunoprecipitation. Optimal isolation of the complex including the PDGFR was obtained using mild lysis conditions with Lubrol, a detergent used for the solubilization of transmembrane protein complexes [17], [18], [19]. Antibodies against the PDGFR co-precipitated both the co-receptor β1-integrin and the ERM proteins following PDGF stimulation (Figure 3A). In addition, the PDGF-activated complex also contained Ras (Figure 3D), suggesting that SOS, being a Ras regulator, may also be part of the complex. Indeed, antibodies against SOS, as well as antibodies against the PDGFR, precipitated the multiprotein complex including either endogenous or overexpressed ERM protein(s) (Figure 3B,C,D). These results demonstrate that both SOS and Ras are part of the complex. Ezrin R579A, however, could not be co-precipitated with SOS or the PDGFR (Figure 3C,D), and, consequently, expression of ezrin R579A blocked the formation of the PDGFR complex.

### Direct interaction of ezrin and Ras is essential for Ras activation

**Figure 4:**
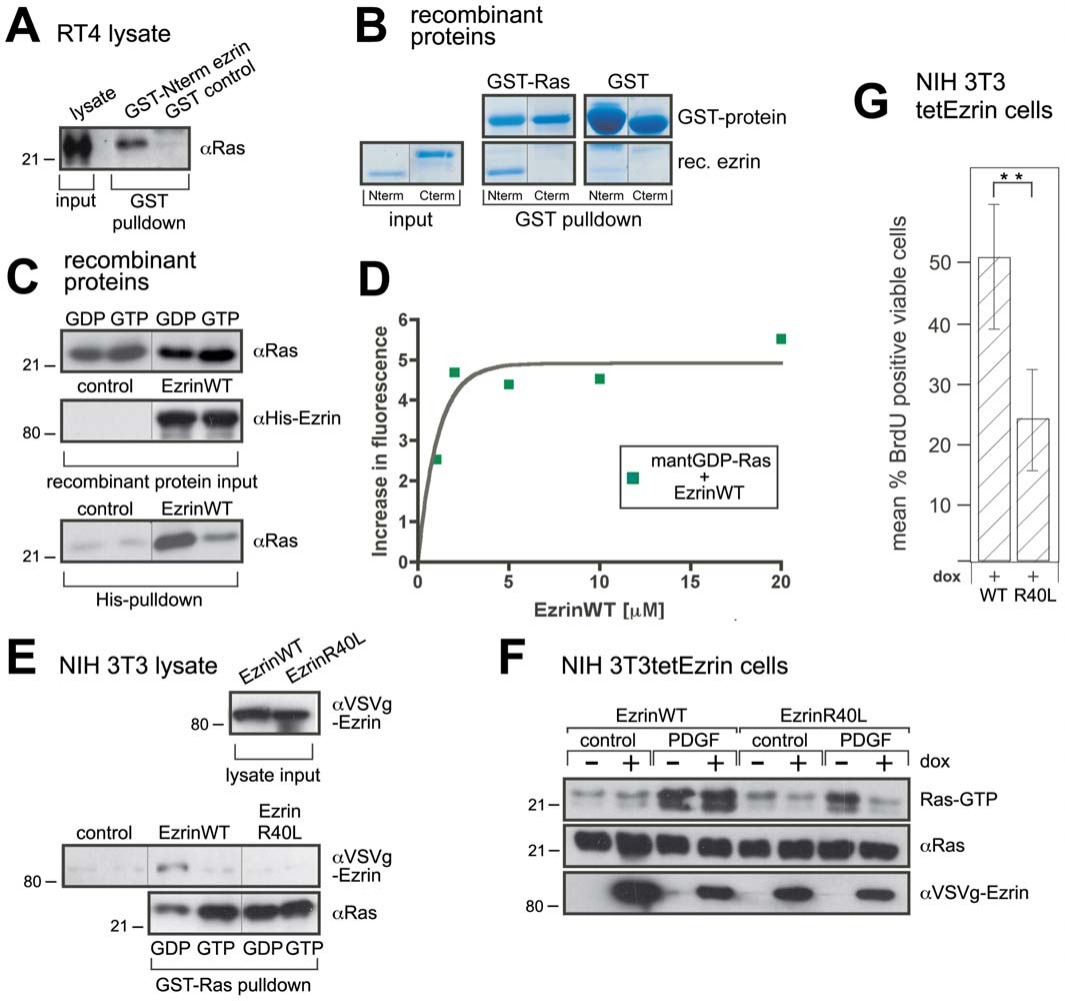
Ezrin interacts directly with Ras. **A**, EzrinNterm interacts with Ras. RT4 cell lysates were incubated with GST-Nterminal ezrin or GST alone. GST pull-downs were immunoblotted as indicated. **B**, Direct interaction between Ras and ezrin determined by GST pull-down. Purified GST-Ras or GST agarose alone was incubated with purified recombinant N-terminal or C-terminal half of ezrin. The pull-down was subjected to SDS-PAGE, followed by colloidal Coomassie staining. **C**, Ezrin binds to GDP-loaded Ras. Recombinant Ras loaded with GDP or non-hydrolysable GTPγS was incubated with His-ezrin wild type. The His pull-down was immunoblotted as indicated. **D**, Ezrin binds fluorescent GDP-Ras in solution. Bacterially expressed Ras was loaded with fluorescent mantGDP. 1 µM mantGDP-Ras was then incubated with increasing amounts of recombinant ezrin wild type and fluorescence intensity was measured. **E**, Ezrin R40L cannot bind Ras. NIH 3T3 lysates of cells expressing dox-inducible ezrin wild type-VSVg or ezrin R40L-VSVg were incubated with GDP- or GTPγS-loaded GST-Ras agarose or GST agarose alone (control). The GST pull-down was immunoblotted as indicated. **F**, Ezrin R40L inhibits PDGF-dependent Ras activation. NIH 3T3 cells expressing dox-inducible ezrin wild type or R40L mutant were plated at a low density, serum starved overnight prior to treatment with PDGF for 3 min. Ras activity was determined as in **1D**. Lysates were immunoblotted as indicated. **G**, Ezrin R40L, but not wild type ezrin, inhibits BrdU incorporation as quantified with a fluorescein-coupled BrdU antibody. Dox-inducible ezrin wild type- or mutant-expressing NIH 3T3 cells were generated and placed in soft agar (− and + dox). Quantitative results represent mean ± s.d. of at least three independent experiments, **P<0.01 using student's t-test. The results are representative of at least three independent assays and each panel represents experiments from the same blot and the same exposure.

Because the association of Ras with SOS should be transient and not stable enough for immunoprecipitation, the co-immunoprecipitation data might indicate that ezrin forms a stabilizing scaffold that assembles Ras and SOS. Employing three independent techniques, we provide evidence for a direct interaction between Ras and ezrin (Figure 4). First, a fusion protein of the N-terminus of ezrin and GST, but not GST alone, brought down Ras from cell lysates (Figure 4A). Furthermore, a fusion protein of GST and Ras precipitated the purified N-terminus of ezrin, but not the C-terminus (Figure 4B). Finally, purified His-tagged ezrin precipitated purified Ras (Figure 4C).

Second, if the interaction of ezrin and Ras serves to stabilize the complex with SOS prior to nucleotide exchange, then Ras-GDP should be the preferred interaction partner of ezrin. Indeed, it was Ras-GDP that was pulled down with His-tagged ezrin (Figure 4C). Another technique using Ras loaded with fluorescent-labeled GDP (mantGDP-Ras) also demonstrated interaction. The fluorescence intensity increased in a concentration-dependent manner when ezrin was added to mantGDP-Ras (Figure 4D), indicating that Ras-GDP and ezrin do indeed interact.

Finally, ezrin carries a motif in its N-terminal domain which resembles the Ras-binding domain of Raf1 kinase [20]. To test whether this motif is involved in the ezrin-Ras interaction, we mutated several amino acids individually within this motif [21], [22]. Of these mutants, ezrin R40L lost the ability to bind Ras most strongly (Figure 4E). Expression of ezrin R40L abolished PDGF-induced activation of Ras (Figure 4F) and inhibited serum-dependent incorporation of BrdU (Figure 4G). Therefore, the most straightforward explanation of these findings is that Ras and ezrin interact directly and that this interaction is part of the scaffolding function of ezrin required for Ras activation.

### Ezrin interacts with the Dbl homology (DH)-pleckstrin homology (PH) domains of SOS

**Figure 5:**
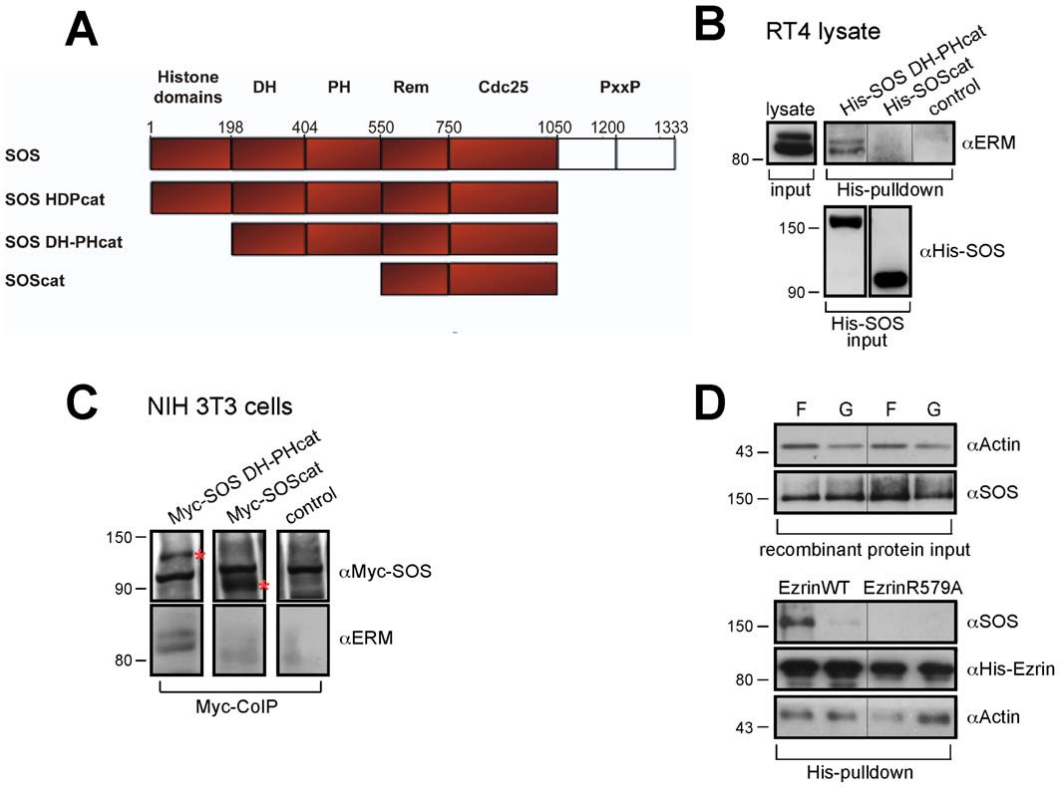
Ezrin interacts with the DH-PH domains of SOS. **A**, Schematic representation of the architecture of used SOS constructs. **B**, ERM proteins interact with the DH-PH domains of SOS. Incubation of His-SOS DH-PHcat- or His-SOScat with RT4 lysates. His pull-downs were immunoblotted as indicated. **C**, ERM proteins interact with the DH-PH domain of SOS. NIH 3T3 cells were transfected with constructs expressing Myc-tagged fragments of SOS (specific band marked with a red asterisk) or an empty vector control. Co-precipitated proteins were immunoblotted with antibodies against the ERM proteins. **D**, Full-length SOS interacts with ezrin *in vitro* only in the presence of F-actin. SOS was incubated with ezrin wild type- or R579A-His in the presence of filamentous (F) or globular (G) actin. The His pulldown was immunoblotted as indicated. The results are representative of at least three independent assays and each panel represents experiments from the same blot and the same exposure.

The ezrin mutants identify ezrin as an organizer of both SOS and Ras. The ability of ezrin to act as a scaffold for SOS was further suggested from mapping the domains of SOS interacting with ezrin (SOS constructs are illustrated and described in Figure 5A). Co-immuno and co-affinity precipitation experiments identified the DH-PH tandem domains as an interaction site for ezrin (Figure 5B,C). As suggested from the co-IP experiments of Figure 3 and ezrin mutant R579A lacking binding to actin, purified recombinant proteins form a trimetric complex of ezrin with SOS (full length) and F-actin, not G-actin (Figure 5D). Wild type ezrin engaged SOS (full length) only in the presence of F-actin, the mutant defective in F-actin binding (R579A) did not (pulldown with His-tagged ezrin). Thus, interestingly, ezrin in conjunction with F-actin appears to adopt a specific SOS-binding conformation, enabling the interaction with the DH/PH domain of SOS.

### Ezrin interaction with the allosteric site of SOS enhances SOS activity

The GEF SOS is itself subject to complex regulation. The DH-PH domains decrease catalytic activity by folding back onto the catalytic domain and restricting the accessibility to a second Ras-binding site distal to the catalytic one. This allosteric Ras-binding site is absolutely required for the full activation of SOS, implicating Ras itself as an essential determinant of SOS regulation [23]. While we identified ezrin as a scaffold protein assembling for both Ras and SOS, we considered whether ezrin might, in addition, help to activate SOS, perhaps by removing this steric block and presenting Ras-GDP to the allosteric site on SOS, essential for SOS activity [23]. This interesting possibility encouraged us to measure cellular SOS activity in the presence or absence of ezrin. Furthermore, *in vitro* experiments comparing SOS with SOS in complex with ezrin were subsequently performed.

**Figure 6:**
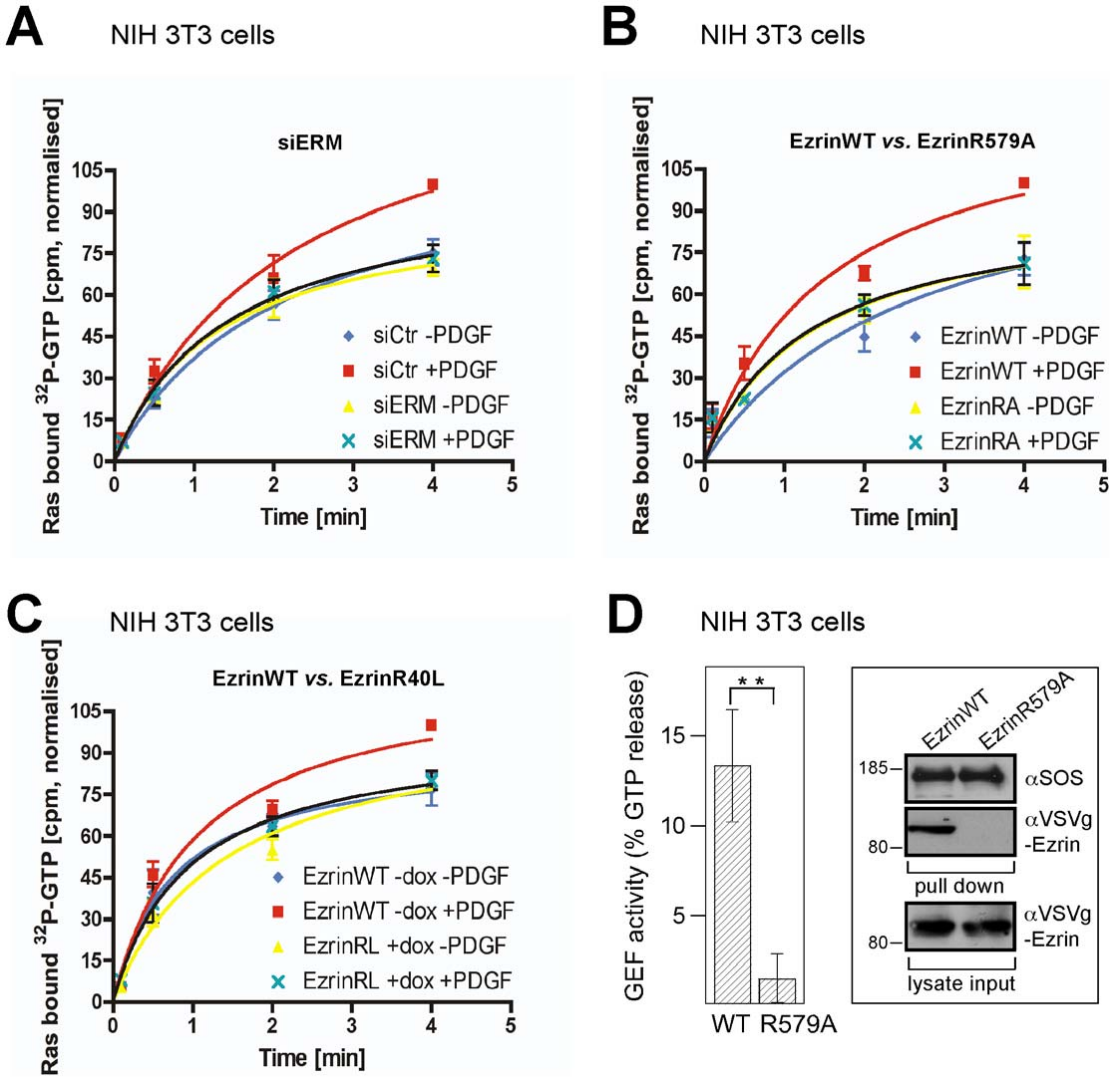
ERM proteins influence full-length SOS GEF activity towards Ras. **A**, *In vivo* GEF activity is associated with presence of ERM proteins. NIH 3T3 cells were treated with siERM cocktail, followed by permeation with streptolysin O in the presence of [α^32^P]-GTP and PDGF treatment. Bound [α^32^P]-GTP caught in the binding pocket of immunoprecipitated Ras was measured. **B** *In vivo* GEF activity is associated with wild type but not R579A ezrin. Dox treatment of dox-inducible ezrin wild type (ezrinWT) or R579A (ezrinRA) NIH 3T3 cells, followed by *in vivo* GEF activity measurement as described in **C**. **C**, *In vivo* GEF activity is associated with wild type but not R40L ezrin indicating that GEF activity is dependent on Ras binding of ezrin. Dox treatment of dox-inducible ezrin wild type (ezrinWT) or R40L (ezrinRL) NIH 3T3 cells, followed by *in vivo* GEF activity measurement as described in **C**. Results represent mean ± s.d. of at least 3 independent experiments. ONEway ANOVA statistical analysis was performed. **D**, SOS/wild type ezrin complex has high *in vitro* GEF activity. (Left panel) SOS/ezrin complexes incubated with Ras preloaded with [α^32^P]-GTP. The reaction was initiated with excess GTPγS and the loss of Ras-bound radioactive GTP measured. Quantitative results represent mean ± s.d. of at least 3 independent experiments, **P<0.01 using student's t-test. (Right panel) Immunoblot shows equal amounts of SOS precipitated from ezrin wild type- and R579A-expressing cells. The results are representative of at least three independent assays and each panel represents experiments from the same blot and the same exposure.

First, we tested whether ezrin is required for SOS nucleotide exchange activity *in vivo* by following the uptake of [α^32^P]-GTP into permeated cells and the loading of [α^32^P]-GTP onto Ras. The nucleotide exchange activity in resting cells was high; however, it was insensitive to ERM protein knockdown and also to the expression of ezrin mutants R579A or R40L, indicating that basal SOS activity does not depend on ERM proteins (Figure 6A–C). At present, we do not have an explanation for this high SOS activity in resting cells, which has also been reported by another group [24]. In marked contrast to the basal SOS activity, the PDGF-sparked acceleration of nucleotide uptake onto Ras was completely abrogated by ERM protein knockdown and also by the expression of ezrin R579A or R40L (Figure 6A–C). The disrupting effect of these ezrin mutants reflects an essential contribution of the ezrin/F-actin and ezrin-Ras complexes to the growth factor-dependent enhancement of SOS activity. We conclude that the ERM proteins are targeted to the plasma membrane/F-actin interface where they assemble SOS and Ras. This action possibly contributes to the release of SOS autoinhibition, thus enabling the presentation of Ras to the allosteric regulatory site of SOS.

### Ezrin controls SOS activity *in vitro*

Based on these *in vivo* data, we investigated the effect of ezrin on SOS activity *in vitro* using precipitated complexes. PDGF-induced protein complexes were collected by GST-Grb2 pulldowns (the stoichiometric interaction of Grb2 with SOS ascertained equal SOS levels) of total lysates of cells expressing wild type or R579A ezrin. The complexes were incubated with recombinant Ras preloaded with radioactive GTP; the exchange reaction was initiated by adding an excess of non-radioactive GTPγS. Despite the presence of equal amounts of SOS (Figure 6D, right panel), a much higher exchange activity was observed in samples containing the SOS-ezrin wild type complex compared with the complexes precipitated from ezrin R579A-expressing cells (Figure 6D). These latter precipitates contained no ezrin (Figure 6D, right panel). This *in vitro* experiment demonstrates that SOS exerted significant nucleotide exchange activity only when bound to wild type ezrin.

## Discussion

We show here that the activation of Ras is linked to actin dynamics and the presence of the ERM proteins. The classic model for the regulation of Ras by the GEF SOS involves the local assembly of SOS through adaptor proteins upon ligand stimulation of a RTK. One of these adaptor proteins, Grb2, brings SOS into proximity with the receptor where it is thought to engage Ras. However, we can now demonstrate a novel step in the control of Ras where regulators of the plasma membrane-cytoskeleton interphase, the ERM proteins, are essential for Ras activation downstream of RTK activity. We show that the ERM proteins in conjunction with F-actin associate with co-receptors, such as β1-integrin, focusing the ERM proteins to relevant sites of RTK activity with which the co-receptors are linked. Our observation that disruption of the interaction of the ERM proteins with co-receptors (by sequestering, by siRNA down-regulation or expression of dominant-negative mutants of the ERM proteins) abolishes growth factor-induced Ras activation, strongly argues for a role of the ERM proteins in the control of Ras.

In response to the growth factor PDGF we could show that the ERM/actin catalyze the formation of a multiprotein complex consisting of RTK, co-receptor, Grb2, SOS and Ras. In addition we identify ezrin and possibly other ERM proteins as new intracellular scaffold partners for Ras and SOS. Ezrin directly assembles Ras and SOS via separate domains of ezrin, and ezrin mutants unable to interact with Ras or SOS severely compromise Ras activity induced by PDGF or EGF. Moreover the PDGF-induced stimulation of guanine nucleotide uptake by Ras in permeated cells is specifically aborted by siRNA down-regulation of the ERM proteins or expression of dominant-negative ezrin mutants. These data suggest that functional ezrin directly assembling Ras and SOS is required for the PDGF-dependent stimulation of nucleotide exchange on Ras.

**Figure 7:**
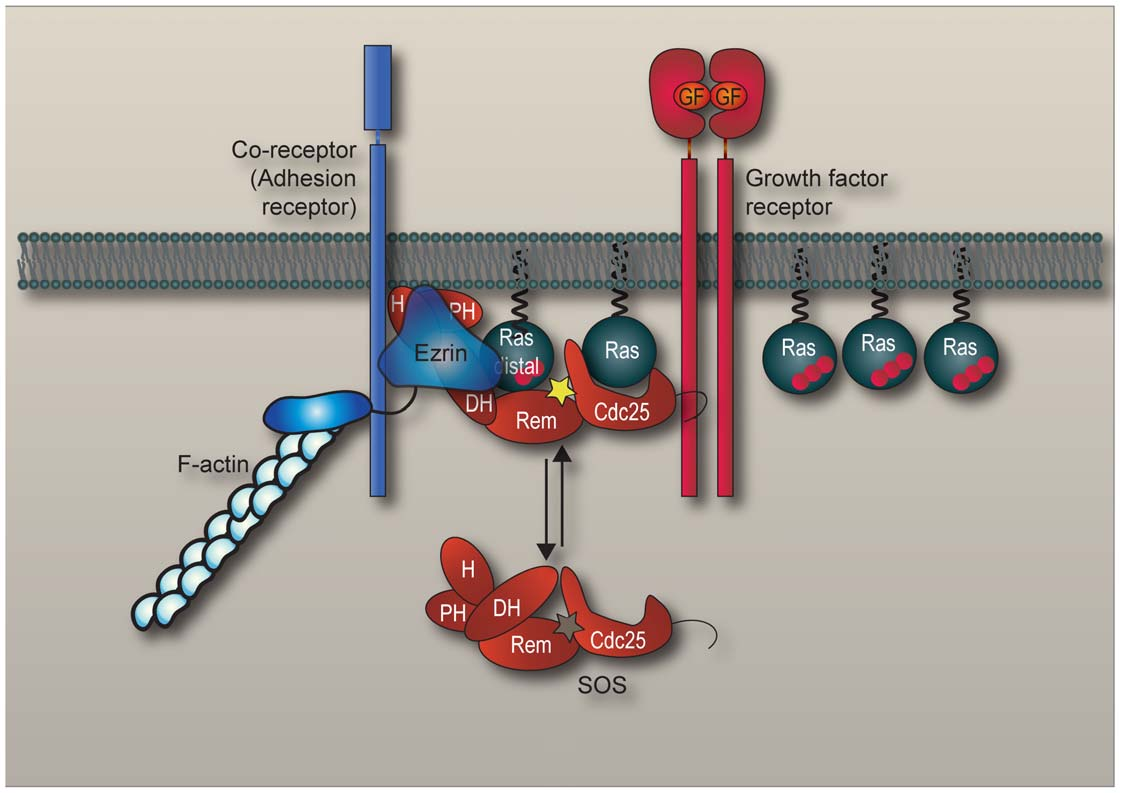
Model of ezrin-mediated activation of SOS. Growth factor (GF) induction promotes ezrin activation, which localizes to co-receptors/adhesion receptors, while SOS couples to the activated RTK. Ezrin in conjunction with F-actin binds to the DH-PH domains of SOS, creating a stable anchorage to the membrane and assisting the release of SOS autoinhibition. With the unmasking of the distal/allosteric site (unmasked site now illustrated by yellow star; masked conformation in grey), ezrin binding to Ras-GDP increases the ability of Ras-GDP to engage this allosteric site, promoting the ability of SOS to catalyze nucleotide exchange (GDP illustrated by two and GTP by three red balls).

Beyond scaffolding function ezrin could be a new intracellular partner required for SOS regulation. SOS, like other GEFs, is a large protein with autoinhibitory domains. According to mutant data [23], SOS requires to be unfolded in order to enable the access of Ras to an allosteric regulatory site which then releases the catalytic activity. The catalytic domain then promotes the exchange of GTP for GDP on another Ras molecule. An *in vitro* Ras activation assay performed with purified SOS isolated from cells demonstrated that its GEF activity is enhanced only when associated with ezrin. We propose that ezrin not only assembles Ras and SOS via separate domains of ezrin, but it interacts with the autoinhibitory DH-PH domains of SOS possibly contributing to the release of SOS autoinhibition and/or presenting Ras to the allosteric regulatory site of SOS (a model illustrating the proposed mechanism of SOS activation is shown in Figure 7).

While this study presents evidence that ezrin is required for the activation of SOS; we should however, consider that other partners are required for the full activation of SOS. A likely candidate is the plasma membrane due to its proximity. Indeed, our *in vitro* GEF assay with isolated SOS contained lipids in addition to ezrin. Lipid interaction has previously been shown to release SOS autoinhibition. For example, binding of phosphoinositides to the PH domain [1] as well as binding of phosphatidic acid to the histone-like domain of SOS are important modulators of its autoinhibition [2], [25], [26]. Taking these findings together, the *in vivo* targeting of ezrin to the plasma membrane and its direct interaction with SOS are both essential features for the full activation of SOS (see proposed model, Figure 7). It is noteworthy to mention that these principles of ezrin-mediated activation of SOS may have a more widespread significance. As several other GEFs also require release from autoinhibition, ezrin might also promote the activation of these GEFs. Indeed, binding of GEFs other than SOS, such as Dbl and Dock180, to ERM proteins has been reported by several laboratories [27], [28], [29]. However, these groups did not address the mechanism of action.

Another feature of ezrin that is required for the activation of Ras is the binding of F-actin. This is demonstrated by the dominant-negative effect of ezrin R579A (which eliminates the binding of F-actin whilst retaining the association with membrane proteins) and by the similar effect of an actin polymerization inhibitor. The most straightforward explanation of these findings is that F-actin is part of a stabilizing complex, which specifically focuses the ERM proteins to RTKs at the plasma membrane. However, our *in vitro* data showing that ezrin can only interact with SOS in the presence of F-actin imply more than a scaffold function: ezrin might adopt a specific SOS-activating conformation only when associated with F-actin. While our findings suggest a role for the ezrin-actin link in signaling, another interesting but different function of actin in signaling relates to the activation of the transcription factor SRF. The abundance of free G-actin inhibits SRF activity by retaining one of its co-activators, MAL, in the cytoplasm [30]. Signaling pathways inhibiting F-actin depolymerization or promoting filament assembly co-operate in preventing the activation of the SRF/MAL complex – this complex addresses a subset of SRF target genes, e.g. the actin promoter itself [31]. Another subset requiring the interaction of SRF and the co-activator TCF is addressed by Erk [32] and is likely to be dependent on the signal-promoting effect of cortical F-actin presented here.

The coupling of co-receptors to ezrin/F-actin could be regarded as a platform to co-ordinate and restrict the encounter of SOS with Ras, thereby fine-tuning growth factor signals. The control of Ras activity is critical and can falter by mutation at several levels, e.g. oncogenic mutations of Ras [33] or an inactivating mutation of the gene *NF1* encoding neurofibromin [34]. In addition, dysregulation of RTKs is often the cause of the development and maintenance of cancer [35]. It is of no surprise that the components of the RTK-dependent signaling steps identified here, such as co-receptors and the ERM proteins, are often overexpressed in cancer [7], [36], [37], [38]. Thus, our proposed model of ezrin-mediated activation of SOS now allows us to conceive how such inappropriate activation of co-receptors, e.g. mis-expression of the co-receptor CD44v6 for the RTK c-Met [39], as well as elevated expression of ezrin [37], radixin [38] and moesin [36] may contribute to cancer progression and metastasis.

## Methods

### Growth factors, antibodies and reagents

Recombinant human platelet-derived growth factor BB (PDGF) (Biomol); recombinant human interleukin-6 (IL-6), epidermal growth factor (EGF), lysophosphatidic acid (LPA), 12-o-tetradecanoyl-phorbol-13-acetate (TPA), Igepal CA-630, Triton-X-100, GDP, GTPγS and doxycycline (dox; Sigma); mantGDP, mantGTP and GST protein (Jena Bioscience); Lubrol 17A17 (Uniqema); ATP (Roche); GST-Grb2 glutathione agarose (GST-Grb2), Raf1-Ras-binding domain glutathione agarose (GST-Raf1-RBD), GST-Ras- and GST-agarose (Upstate); latrunculin B (Calbiochem); glutathione agarose (Santa Cruz). Antibodies: GST (B-14), Integrin-β1 (M-106), Erk1 (K23), Ezrin (C-19), moesin (C-15), radixin (C-15), Actin (I-19), SOS1 (C-23), PDGFR-β (958), GFP (B-2), Myc (9E10), Tubulin (TU-02) (Santa Cruz); Ras (RAS10) (Upstate); VSVg, HA (Roche); Flag (F1804) (Sigma); ezrin (3C12) (NeoMarkers); against phosphorylated proteins: PDGFR-β (Y716) (Upstate); Erk (Thr202/Tyr204), Stat3 (Tyr705), CREB (Ser133), ezrin(Thr567)/radixin(Thr564)/moesin (Thr558) (Cell Signalling).

### Plasmids

EzrinWT-, N- and Cterminal-GFP, and ezrin(ΔABD)-EGFP-N1 (Antonio Sechi, Braunschweig). EzrinWT- and T567D-VSVg (pCB6), ezrin N- and Cterminal-GST, Human ezrinWT- and T567D-Myc in pcDNA3.1 (Monique Arpin, Paris). EzrinWT- and T567D-VSVg fragments were subcloned into pUHD10.3 rtTA-responsive cloning vector (EcoR1/Xba1). Ezrin Nterm- and Cterm-GST were cloned into pGEX4T-1 vector (BamH1/EcoR1). Human moesinWT (Dominique Lallemand, Paris) expression construct was generated in pcDNA3.1 (Kpn1/Not1), and a dox-inducible radixinWT-Flag (pcDNA3 Human radixinWT-Flag, David Gutmann, St. Louis, Washington) was created in pUHD10.3 (BamH1/EcoR1). Myc-SOS and His-SOScat constructs were generated by PCR, then cloned into the pcDNA3.1/V5-His-TOPO vector (Invitrogen). GST-CD44tail and GST-CD44tail mutant in pEBG-GST were generated as described in Morrison *et al.*, 2001. Myc-H-Raps and HA-RasWT in pcDNA3.1 (UMR cDNA Resource Center, University of Missouri-Rolla, USA). For protein purification, ezrinWT-His was cloned in pET15TEV expression vector (C. Breithaupt-Than, Halle). His-SOScat (aa 564–1094) in pProEX HTb expression vector was obtained from A. Wittinghofer, Dortmund. His-SOS DH-PHcat (aa 198–1049), His-SOS HDPcat (aa 1–1049) and His-H-Ras (aa 1–166) were cloned in pET15TEV expression vector (BamH1). All point mutations were generated using a site-directed mutagenesis kit (Stratagene).

### siRNA experiments

The down-regulation of ERM proteins was carried out using short interfering RNA (siRNA) according to the procedure described in [40]. Single siRNA oligonucleotides were purchased from Ambion (only the sense strand is reported): ezrin (rat, Set I): 5′-UCAACUAUUUCGAGAUCAAAA-3′ (ORF 606–629), ezrin (rat, Set II): 5′-GGGACUCAGGCCUGUUUAUtt-3′ (ORF 2710–2732); radixin (rat, Set I): 5′-CUCGUCUGAGAAUCAAUAAGC-3′ (ORF 752–775), radixin (rat, Set II): 5′-GCUGUGGCUGGGCGUUGAUtt-3′ (ORF 852–874); moesin (rat, Set I): 5′-GGCUGAAACUCAAUAAGAAGG-3′ (ORF 171–194), moesin (rat, Set II): 5′-GGCCCUGCUGCAGGCUUCUtt-3′ (ORF 1299–1321); ezrin (mouse, Set I): 5′-GCCGUAUGUAGACAAUAAAGG-3′ (ORF 139–161), ezrin (mouse, Set II): 5′-GGCCAAGUUCGGAGAUUAUtt-3′ (ORF 528–550); radixin (mouse, Set I): 5′-GCACCUCGUCUGAGAAUCAAU-3′ (ORF 809–822), radixin (mouse, Set II): 5′-GCUGUGGCUAGGUGUUGAUtt-3′ (ORF 1195–1217); moesin (mouse, Set I): 5′-GCAAGCCUGACACCAUUGAGG-3′ (ORF 882–905), moesin (mouse, Set II): 5′-GGCCCUGCUGCAGGCUUCUtt-3′ (ORF 1388–1410); Control (Ctr) siRNA (against firefly luciferase) 5′-CGUACGCGGAAUACUUCGA-3′. SMART pools (cocktail of four siRNAs) against ezrin (mouse), moesin (mouse) and radixin (mouse) were purchased from Dharmacon (sequences will be provided upon request). The down-regulation of ERM proteins in HeLa and RPM-MC was carried out the following siRNA duplexes: ezrin (human, Set I): 5′-CCCCAAAGAUUGGCUUUCC-3′ (ORF 704–722), ezrin (human, Set II); 5′-UCCACUAUGUGGAUAAUAA-3′ (ORF 140–158); moesin (human, Set I): 5′-AAAAGCCCCGGACUUCGUC-3′ (ORF 786–804), moesin (human, Set II): 5′-AGAUCGAGGAACAGACUAA-3′ (ORF 1058–1076); radixin (human, Set I): 5′-GCAGUUGGAAAGGGCACAA-3′ (ORF 948–966), radixin (human, Set II): 5′-CUCGUCUGAGAAUCAAUAA-3′ (ORF 752–775). As control, the following scrambled duplexes (derived from Set I duplexes) were used: ezrin 5′-CCGUCACAUCAAUUGCCGU-3′, moesin 5′-ACUAGACGAACCGUCGCUC-3′, radixin 5′-GAUGCAGCAGCAUGAAGAG-3′.

### Cell culture

RT4-D6P2T (RT4) rat schwannoma cell line ENU-induced RT4-D6P2T rat schwannoma cell line (mutated *Erb*B2 activates the Ras-MAP kinase cascade) [41], NIH 3T3 mouse fibroblasts and HeLa human cervix epitheloid carcinoma cell line (European Collection of Animal Cell Cultures). RT4 and NIH 3T3 cells carrying doxycycline (dox)-inducible ezrin wild type or ezrin mutants (named either RT4tetEzrin or NIH 3T3tetEzrin) were prepared as described in Morrison *et al.*, 2001. Cells were grown in Dulbecco's modified Eagle's medium (DMEM; Gibco-BRL) supplemented with 10% foetal calf serum (FCS; Gibco-BRL) or 10% donor calf serum (Gibco-BRL) for NIH 3T3. All cells were maintained in a humidified atmosphere with 5% CO_2_ at 37°C. The concentration of dox in all cell culture experiments was 1 µg/ml.

### Stable and transient transfection of cells

Transfections (both RNAi and plasmid DNA) were performed using Lipofectamine (Invitrogen), in accordance with the manufacturer's instructions. To generate stable or pooled clones, cells were co-transfected with pCEP4 (hygromycin resistance) (Invitrogen), and selected in 100 µg/ml hygromycin (Roche). Note, dox-inducible ezrin wild type or ezrin mutants as well as ezrin expressing stable/pooled clones over-expressed exogenous ezrin more than 35 times compared to endogenous ezrin levels.

### Definition of growth condition in culture dishes

Low cell density ( = logarithmic or exponential growth) was defined as the density recorded at 24 h after seeding 500 cells per cm^2^. High cell density ( = confluent growth condition) was defined as the density recorded at 24 h after seeding 5×10^3^ cells per cm^2^ (1×10^5^ cells per cm^2^ for NIH 3T3).

### Fluorescence microscopy

Cells were grown on coverslips placed in six-well plates. Cells were fixed with 4% paraformaldehyde in “cytoskeleton buffer” (CB: 10 mM PIPES, 150 mM NaCl, 5 mM EGTA, 5 mM glucose, 5 mM MgCl2, pH 7.0) at room temperature for 20 min, washed 3 times in CB and permeated in 0.05% Triton-X-100 in PBS for 1 min. Actin fibers were detected by phalloidin conjugated to Alexa 594 (Molecular Probes) for 1 hour at a dilution of 1∶50. Cells were washed 3 times, mounted on glass slides with 10 µl Prolong Antifade reagent (Molecular Probes) and images obtained using epifluorescence with an Axiovert 135 TV microscope (Zeiss) equipped with a Plan-Apochromat 100×/1.40 NA oil immersion objective. Images were recorded with a cooled, back-illuminated CCD camera (TE/CCD-1000 TKB, Princeton Instruments) driven by IPLab Spectrum software (Scanalytics).

### GEF activity measurement – streptolysin O

Intracellular GEF activity was measured as described in de Vries-Smits *et al.*, 1995 and Stephens *et al.*, 1993. In brief, NIH 3T3 cells were seeded in 6-well plates at 6×10^3^ cells/cm^2^. Transfection of siRNA duplexes or DNA constructs expressing ezrinWT, R579A was performed the next day (the R40L a dox inducible mutant cell line was used). Another day later, cells were deprived of serum overnight to perform the assay after 48 h of ERM knock-down or construct expression (dox added were indicated). Assay kinetics was started after culture medium was replaced with 600 µl permeation buffer (50 mM HEPES pH 7.5, 107 mM K-glutamate, 23 mM NaCl, 3 mM MgCl_2_, 0.3 mM CaCl_2_, 1 mM EGTA, 1 mM ATP, 30 U/ml streptolysin O (Aalto Bioreagents), 100 µCi/ml [α^32^P]-GTP (Hartmann Analytik) and −/+ 10 ng/ml PDGF). At indicated time points, the exchange reaction was stopped by adding 1 ml lysis buffer (50 mM HEPES pH 7.5, 100 mM NaCl, 10 mM MgCl_2_, 1% NP-40, 100 µM GDP, 100 µM GTPγS, protease inhibitor cocktail ‘complete w/o EDTA’ (Roche) and 5 µg/ml anti-Ras antibody Y13-259). Lysates were cleared by centrifugation, supplemented to contain 500 mM NaCl, 0.6% sodium deoxycholate and 0.06% SDS, and Ras was immunoprecipitated with protein Gplus agarose (Oncogene). The precipitates were washed six times with 1 ml Ras wash buffer (50 mM HEPES pH 7.5, 500 mM NaCl, 5 mM MgCl_2_, 0.1% Triton-X-100 and 0.005% SDS) and subjected to Cherenkov counting.

### GEF activity measurement – SOS containing complexes from cells

Measurement of nucleotide exchange activity of SOS towards Ras was performed as described in Downward *et al.*, 1995 and Rubio *et al.*, 1999. In brief, four 15 cm plates were used. In each plate 2×10^6^ dox inducible NIH 3T3 cells were seeded (dox inducible for ezrinWT or ezrinR579A mutant). The next day cells were serum starved and dox added overnight. Following day, in each plate cells were lysed in 1 ml lysis buffer after 10 ng/ml PDGF stimulation (50 mM HEPES pH 7.4, 0.5% Lubrol 17A17, 100 mM NaCl, 5 mM sodium vanadate, and protease inhibitor cocktail ‘complete w/o EDTA’). The lysate was mixed and then divided again into four and SOS was pulled down for 2 h using GST-Grb2. Equal amount of SOS and the presence of ezrin were confirmed by immunoblotting. The pulldowns were stored on ice until used in the exchange assay. ***Ras loading:*** Recombinant H-Ras (50 ng) was loaded at 37°C for 5 min with 25 µCi [α^32^P]-GTP in 100 µl loading buffer (50 mM HEPES pH 7.5, 5 mM EDTA and 5 mg/ml BSA). The reaction was stopped by adding 5 µl 0.5 M MgCl_2_ on ice. High-magnesium buffer (800 µl) (50 mM HEPES pH 7.5 and 5 mM MgCl_2_) and 1 mM cold, non-radioactive GTP were added, and MgCl_2_ was adjusted to 5 mM. This mixture (25 µl) was added to the immunoprecipitates and nucleotide exchange was assayed at 30°C for 30 min. The reaction was stopped by placing on ice and adding 300 µl Ras wash buffer, 1 mg/ml BSA and 5 µg Y13-259 anti-Ras antibody. Ras was immuno-precipitated with protein Gplus agarose. The precipitates were washed four times with 1 ml Ras wash buffer and subjected to Cherenkov counting.

### Protein purification from *Escherichia coli*

The expression vector pET15TEV fuses an N-terminal polyhistidine tag to the protein followed by a tobacco etch virus (TEV) cleavage site. *E. coli* cells (Rosetta 2 DE3, Novagen) were transformed with SOS, ezrin or Ras protein expression constructs and grown in LB medium supplemented with antibiotics (100 mg/l ampicillin and 100 mg/l chloramphenicol; Carl Roth). Protein expression was induced by 100 µM IPTG (for Ras; Carl Roth), 250 µM IPTG (SOS) or 500 µM IPTG (ezrin) at a cell density corresponding to an absorbance of 0.4–0.6 at 600 nm, and the proteins were expressed at 33°C overnight (Ras), at 30°C for 6 h (SOS) or at 37°C for 4 h (ezrin). Cells were collected by centrifugation for 20 min at 6,000×*g*, resuspended in either Ras resuspension buffer (RB; 25 mM Tris pH 7.4, 2.5 mM MgCl_2_, 100 µM GDP and 0.5% Triton-X-100), SOS resuspension buffer (50 mM Tris pH 7.4, 1 M NaCl and 0.5% Triton-X-100) or ezrin resuspension buffer (50 mM Tris pH 8.0 and 150 mM NaCl) containing protease inhibitors and snap frozen in liquid nitrogen. After thawing, lysozyme (Carl Roth) was added. Cell lysis was carried out by ultrasonification (15 sec impulse, 30-sec break). Cell debris was collected by centrifugation at 15,000×*g* in a JA 25.50 rotor for 10 min. All purifications were carried out at 4°C. The remaining clear lysate was incubated with Ni-Sepharose 6 Fast Flow (GE Healthcare) overnight and transferred to Glass Econo-Column Columns (Bio-Rad). After several washing steps with RB and RB plus 30 mM imidazole, proteins were eluted using 250 mM imidazole in the corresponding RB (including 140 mM NaCl for Ras). Fractions containing proteins were pooled and dialyzed against buffer: Ras (50 mM Tris pH 7.4, 5 mM MgCl_2_ and 1 mM DTT); SOS (20 mM Tris pH 8.0 and 100 mM NaCl); and ezrin (50 mM Tris pH 8.0 and 150 mM NaCl). SOS and Ras proteins were aliquoted, snapped frozen and stored at −80°C. Ezrin proteins were either kept at 4°C or −20°C. When required, the His-tag was cleaved using TEV Protease (Promega) following the manufacturer's instructions.

### Protein purification from insect cells

Full-length SOS1 was prepared essentially as described in [42]. Briefly, Sf9 cells (Merck, Biosciences, Germany) infected in triple-bottom culture flasks with a baculovirus encoding the sequence of full-length human SOS1 in fusion with an N-terminal Glu-Glu epitope tag (EYMPME) (cloned in transfer vector pAcC13, kind gift of Frank McCormick, San Francisco) were harvested 72 h post infection. Cells were washed once in PBS, resuspended in 10 ml ice-cold hypotonic solution (50 mM Tris pH 7.5, 5 mM MgCl_2_, 1 mM DTT and protease inhibitors)/ml cell pellet and placed on ice for 10 min. Cells were lysed with 20 strokes in a Dounce homogenizer on ice and insoluble debris was removed by centrifugation at 100,000×*g* for 30 min at 4°C. The supernatant was loaded onto a 1–2-ml anti-Glu-Glu column pre-equilibrated in buffer A (50 mM Tris pH 7.5, 150 mM NaCl, 5 mM MgCl_2_, 1 mM DTT and protease inhibitors), previously prepared by coupling 1 mg anti-Glu-Glu antibody (kind gift of Julian Downward, London) to 1 ml Actigel matrix (Sterogene) following the manufacturer's instructions. The column was sequentially washed with 10 ml buffer A, 10 ml buffer A supplemented with 0.5% NP-40, 10 ml buffer A supplemented with 500 mM NaCl and 10 ml buffer A. Glu-Glu-SOS was eluted by applying buffer A containing 50 mg/ml EYMPME peptide. SOS fractions were pooled, concentrated, dialyzed against 50 mM Tris pH 7.5, 50 mM NaCl, 1 mM DTT and 50% glycerol, and stored at −20°C. Kept in this way, SOS retained nucleotide exchange activity towards recombinant H-Ras for at least two weeks.

### BrdU-based proliferation assay

NIH 3T3 cells expressing dox-inducible ezrin wild type or R40L mutant were plated at low cell density on coverslips placed in 12-well plates, followed by incubation with 20 µM/well bromodeoxyuridin (BrdU, Oncogene) for 3 h. Cells were fixed with 4% paraformaldehyde in ‘cytoskeleton buffer’ (CB: 10 mM PIPES, 150 mM NaCl, 5 mM EGTA, 5 mM glucose and 5 mM MgCl_2_, pH 7.0) at room temperature (RT) for 20 min, washed three times in PBS and permeated in 0.1% Triton-X-100 in PBS for 1 min. After blocking in 1% BSA for 1 h, the coverslips were treated with a cocktail of 2 µg/ml DAPI (Invitrogen), 250 µg/ml α-BrdU/Fluorescein (Alexis), 10× DNase-Buffer, DNase and 200 µg/ml α-myc/TRITC for 1 h. Cells were washed three times in PBS, mounted on glass slides with 10 µl Prolong Antifade reagent (Molecular Probes) and images obtained using epifluorescence with an Axiovert 135 TV microscope (Zeiss).

### Soft agar assay

RT4 stably transfected cells were detached from culture plates with 0.25% trypsin, resuspended in complete medium and counted. Cells (1×10^4^) were resuspended in 3.6 ml of medium containing 10% FCS. This mastermix was split into two, doxycycline (1 µg/mL) was added to one half and to each mixture 200 µl of warm agar solution (stock is 3.3% in sterile PBS) was added and mixed. The mixture was rapidly dispensed into 24-well plates at 500 µl volume and cooled for 2 min at 4°C. Colonies were counted after 10 to 14 days.

### siRNA experiments

Single siRNA oligonucleotides were purchased from Ambion, used siRNA sequences can be found in the Supplementary methods. In brief 4×10^5^ cells were seeded overnight in a 10 cm plate. Transfection of all siRNAs was performed using lipofectamine according to manufacturer's instructions. The following day cells were given fresh medium and left to recover for at least 8 h. Cells were serum starved overnight prior to 10 ng/ml PDGF stimulation for 5 minutes.

### siRNA with exogenous reconstitution of human ezrin, radixin and moesin

NIH 3T3 cells were transfected with ERM siRNAs using lipofectamine. The following day cells were given fresh medium and left to recover for at least 8 h. Cells were then transfected with plasmid DNA containing human ezrin, radixin and moesin using lipofectamine according to manufacturer's instructions. Cells were serum starved overnight prior to 10 ng/ml PDGF stimulation for 5 min.

### Affinity precipitation and immunoprecipitation

For pull-downs with GST-Raf1-RBD, cells were lysed in 25 mM HEPES pH 7.5, 150 mM NaCl, 10 mM MgCl_2_, 1% Igepal CA-630, 10% glycerol, 1 mM EDTA, 1 mM sodium vanadate, 1 mM PMSF, 10 mg/ml aprotinin and 10 mg/ml leupeptin. For affinity precipitation supernatants were incubated with 5 µg recombinant fusion protein rotating at 4°C for 1 h. Ras assays were performed on pooled clones expressing ezrin wild type compared to ezrin R579A mutant. 4×10^5^ cells were seeded overnight in a 10 cm plate. Cells were serum starved overnight prior to 10 ng/ml PDGF stimulation for 3 min. For pull-downs with GST-Nterminal ezrin or GST-Grb2, cells were lysed in 25 mM HEPES pH 7.5, 20 mM NaCl, 10 mM MgCl_2_, 0.5% Lubrol 17A17, 1 mM sodium vanadate, 1 mM PMSF, 10 mg/ml aprotinin and 10 mg/ml leupeptin. Supernatants were incubated with either 5 µg recombinant fusion protein rotating at 4°C overnight. For all pull-downs 4×10^5^ cells were seeded overnight in a 10 cm plate.

For immunoprecipitation procedure as in Morrison *et al.*, 2001 [16]. In brief, 2×10^6^ cells were seeded overnight in a 20 cm plate. Cells were serum starved overnight prior to 10 ng/ml PDGF stimulation for 1 minute. Cells were lysed in 25 mM HEPES pH 7.5, 20 mM NaCl, 0.5% Lubrol 17A17, 1 mM sodium vanadate, 1 mM PMSF, 10 mg/ml aprotinin and 10 mg/ml leupeptin. Supernatants were incubated with 2 µg of antibody rotating at 4°C for 1 h.

### SDS-PAGE, immunoblotting and coomassie staining

Following electrophoresis with 7.5–12.5% SDS-PAGE gels, the proteins were transferred to PVDF membranes (Millipore) and treated with blocking buffer (5% skimmed milk, 0.1% Tween-20, 10 mM Tris at pH 7.6 and 100 mM NaCl) at RT for 1 h. The membranes were then incubated with primary antibody in blocking buffer for another hour at RT or overnight at 4°C. After three washes, the membranes were incubated with secondary antibody at RT for 1 h, then developed and visualized using enhanced chemiluminescence (Amersham). For coomassie-based gel staining, Imperial protein stain solution (ThermoScientific) was used. Immunoblotting quantification was performed using Quantity One software (Bio-Rad Laboratories). Note: immunoblots for αERM (unless otherwise stated) a mixture of ezrin (C-19), radixin (C-15) and moesin (C-15) was used. For a single ezrin blot, the 3C12 antibody was used.

### Pull-down assays using recombinant proteins

#### Ras/ezrin

Ras/ezrin interaction was tested for different conditions (GTPγS-Ras *vs.* GDP-Ras) and different purified ezrin protein mutants. Ras loading with nucleotides was performed as described in Margarit *et al.*, 2003 [43]. The respective His-tagged ezrin protein (1–2 µg) was linked to Ni-Sepharose and incubated with 50–100 ng loaded Ras in 500 µl interaction buffer (50 mM HEPES pH 7.5, 50 mM NaCl, 10 mM MgCl_2_ and 0.5% Triton-X-100) overnight at 4°C under constant rotation. The following day, the probes were washed four times with 100 µl ice-cold washing buffer (50 mM HEPES pH 7.5, 80 mM NaCl, 10 mM MgCl_2_ and 0.5% Triton-X-100), with a short centrifugation step between the washings. During the final washing step, the washing buffer was completely removed and the remaining proteins were subjected to SDS-PAGE and immunoblotting. ***SOS/ezrin:*** SOS/ezrin interaction studies were performed either in the presence or absence of F-actin/G-actin. G-actin (Cytoskeleton) was polymerized to F-actin following the manufacturer's protocol. Interactions between SOS and ezrin, and F-actin/G-actin were then carried out either in F-actin-Buffer (5 mM Tris pH 8.0, 0.2 mM CaCl_2_, 50 mM KCl, 2 mM MgCl_2_, 1.2 mM ATP and 0.5 mM DTT) or G-actin-Buffer (5 mM Tris pH 8.0, 0.2 mM CaCl_2_, 0.2 mM ATP and 0.5 mM DTT) following the procedure above.

### Fluorescent GEF activity measurements *in vitro*

Nucleotide loading and nucleotide exchange measurements on Ras were performed as described previously in Margarit *et al.*, 2003 [43]. Dissociation rates were measured on a fluorescence spectrometer combined with a stop-flow device (SFM-400; Bio-Logic). Substrate solution containing 1 µM mantGDP-Ras was automatically mixed with reaction buffer supplemented with 200 µM GDP and additional proteins such as SOS and/or ezrin, when indicated. Dissociation was measured for 360 s. The data were fitted either to a linear (Y = I+B*x, with nucleotide dissociation rate = absolute(B)), single exponential (Y = Y_0_+A_1_^−k1*x^) or bi-exponential (Y = Y_0_+A_1_^−k1*x^+A_2_^−k2*x^) function (with I as the intercept, B as slope, A_1_/A_2_ as the amplitude, Y_0_ as the fluorescent value at infinite time, k1/k2 as the apparent dissociation rate constant and x as time) using the program Origin 8Pro.

## Supporting Information

Figure S1 **ERM proteins are necessary for PDGFR signaling.** **A**, Loss of ERM binding to CD44 tail upon point mutation of CD44. CD44 tail fused to GST was over-expressed in RT4 cells kept at low cell density. Cells were lysed, GST-CD44 tail was affinity-precipitated and derived complexes were analyzed by Western blot employing a mixtureof antibodies against ERM proteins and GST for loading. The mutated CD44 tail reveals strongly diminished though not absent ERM binding. **B–D**, Removal of the ERM proteins from the co-receptor at the plasma membrane interfered with signalling. RT4 cells were transfected with a construct expressing either the soluble ezrin-binding domain of CD44 (GST-CD44tail) or the point-mutated domain defective in ezrin binding (GST-CD44tail mutant). Transfected cells were plated at low density,serum starved overnight prior to induction with PDGF (10 ng/mL), lysed and immunoblotted as indicated. **B**, PDGF induced stimulation (5 min) of Erk is attenuated in cells expressing a soluble ERM binding domain (wild type GST-CD44 tail) compared to a mutant version. **C, D**, Kinetic resolution of PDGF induced signalling in cells expressing a soluble ERM binding domain. PDGF induces a sharp incline in Erk phosphorylation within 1 minute in cells expressing a mutant form of the CD44 tail. Phosphorylation levels stay high in the period investigated. Expression of the ERM binding domain (wild type GST-CD44 tail) attenuates ERK activation characterized by shifted phosphorylation maximum (from 1 to 5 minutes) and lower fold-induction (from 19 to 5 times). Quantitation of pErk was performed with ImageJ. **E, F**, Downregulation of ERM protein expression reduces PDGF-dependent Erk phosphorylation. E, RT4 cells were treated with two independent siRNA cocktails against ERM proteins or control siRNA and serum starved overnight prior to induction with PDGF (10 ng/ml, 5 min). Lysates were immunoblotted as indicated. **F**, HeLa cells were plated at low density, treated with a cocktail of siRNAs against all three ERM proteins or control siRNA for 24 h, then serum starved overnight prior to treatment with PDGF (10 ng/ml, 5 min). Lysates immunoblotted as indicated.
(TIF)

Figure S2 **Ezrin R579A mutant inhibits PDGF-induced Erk phosphorylation.** **A**, RT4 cells expressing dox-inducible ezrin wildtype-VSVg were plated at low density, serum starved overnight prior to treatment with either PDGF (20 µM, 5 min), TPA (100 ng/ml, 5 min) or IL-6 (1 ng/ml, 5 min). Lysates immunoblotted as indicated. **B**, RT4 cells expressing dox-inducible ezrin R579A mutant-VSVg were plated at low density, serum starved overnight prior to treatment with either PDGF (20 µM, 5 min), TPA (100 ng/ml, 5 min) or IL-6 (1 ng/ml, 5 min). Lysates immunoblotted as indicated.
(TIF)
