# Supplementary figures and images for: Activation of Ras Requires the ERM-Dependent Link of Actin to the Plasma Membrane
Source: PLoS One. 2011 Nov 21;6(11):e27511. doi: 10.1371/journal.pone.0027511 (PMC3221661; doi:10.1371/journal.pone.0027511)

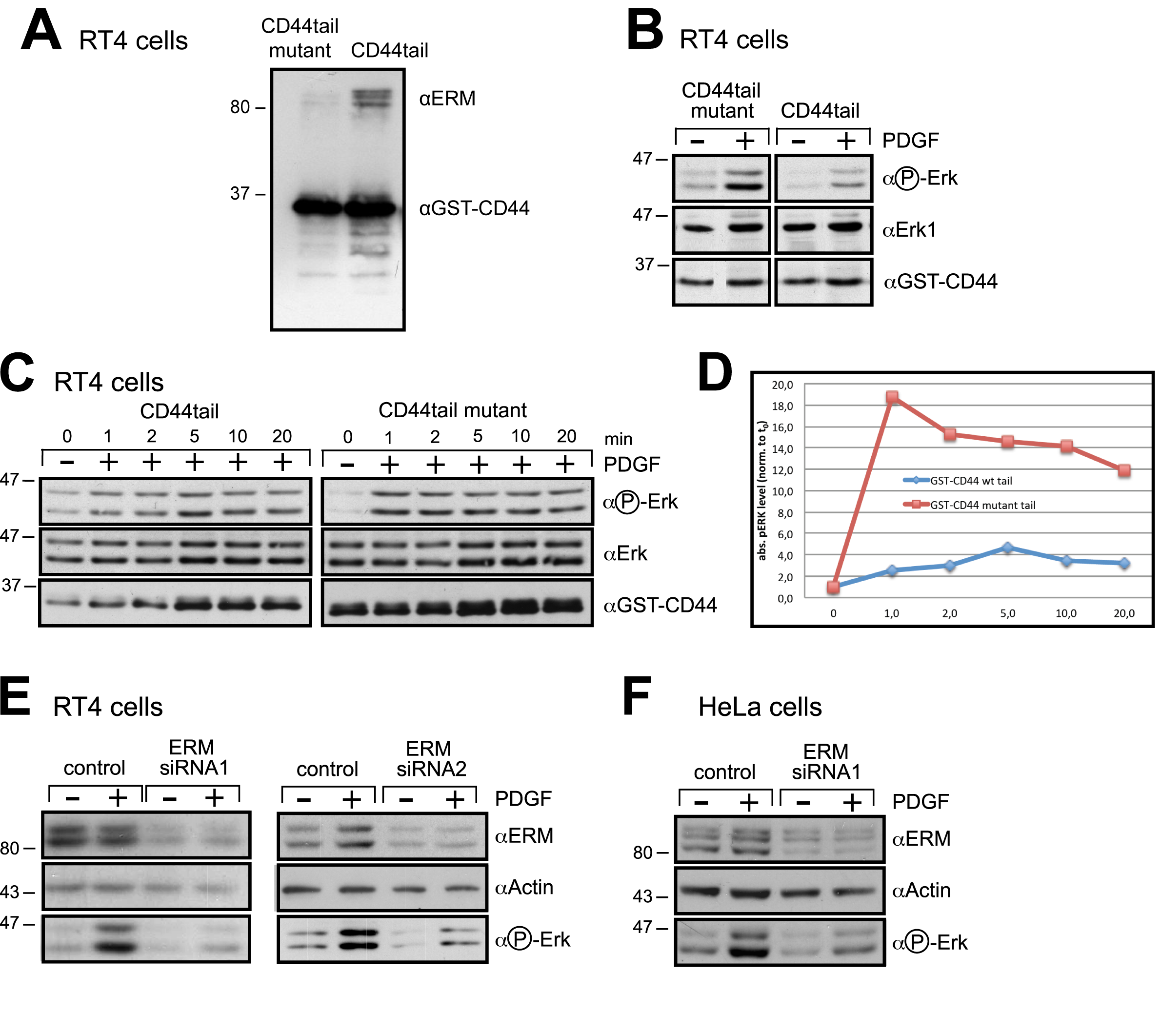

Supplement: Figure S1 — ERM proteins are necessary for PDGFR signaling. A, Loss of ERM binding to CD44 tail upon point mutation of CD44. CD44 tail fused to GST was over-expressed in RT4 cells kept at low cell density. Cells were lysed, GST-CD44 tail was affinity-precipitated and derived complexes were analyzed by Western blot employing a mixtureof antibodies against ERM proteins and GST for loading. The mutated CD44 tail reveals strongly diminished though not absent ERM binding. B–D, Removal of the ERM proteins from the co-receptor at the plasma membrane interfered with signalling. RT4 cells were transfected with a construct expressing either the soluble ezrin-binding domain of CD44 (GST-CD44tail) or the point-mutated domain defective in ezrin binding (GST-CD44tail mutant). Transfected cells were plated at low density,serum starved overnight prior to induction with PDGF (10 ng/mL), lysed and immunoblotted as indicated. B, PDGF induced stimulation (5 min) of Erk is attenuated in cells expressing a soluble ERM binding domain (wild type GST-CD44 tail) compared to a mutant version. C, D, Kinetic resolution of PDGF induced signalling in cells expressing a soluble ERM binding domain. PDGF induces a sharp incline in Erk phosphorylation within 1 minute in cells expressing a mutant form of the CD44 tail. Phosphorylation levels stay high in the period investigated. Expression of the ERM binding domain (wild type GST-CD44 tail) attenuates ERK activation characterized by shifted phosphorylation maximum (from 1 to 5 minutes) and lower fold-induction (from 19 to 5 times). Quantitation of pErk was performed with ImageJ. E, F, Downregulation of ERM protein expression reduces PDGF-dependent Erk phosphorylation. E, RT4 cells were treated with two independent siRNA cocktails against ERM proteins or control siRNA and serum starved overnight prior to induction with PDGF (10 ng/ml, 5 min). Lysates were immunoblotted as indicated. F, HeLa cells were plated at low density, treated with a cocktail of siRNAs agains [file pone.0027511.s001.tif]

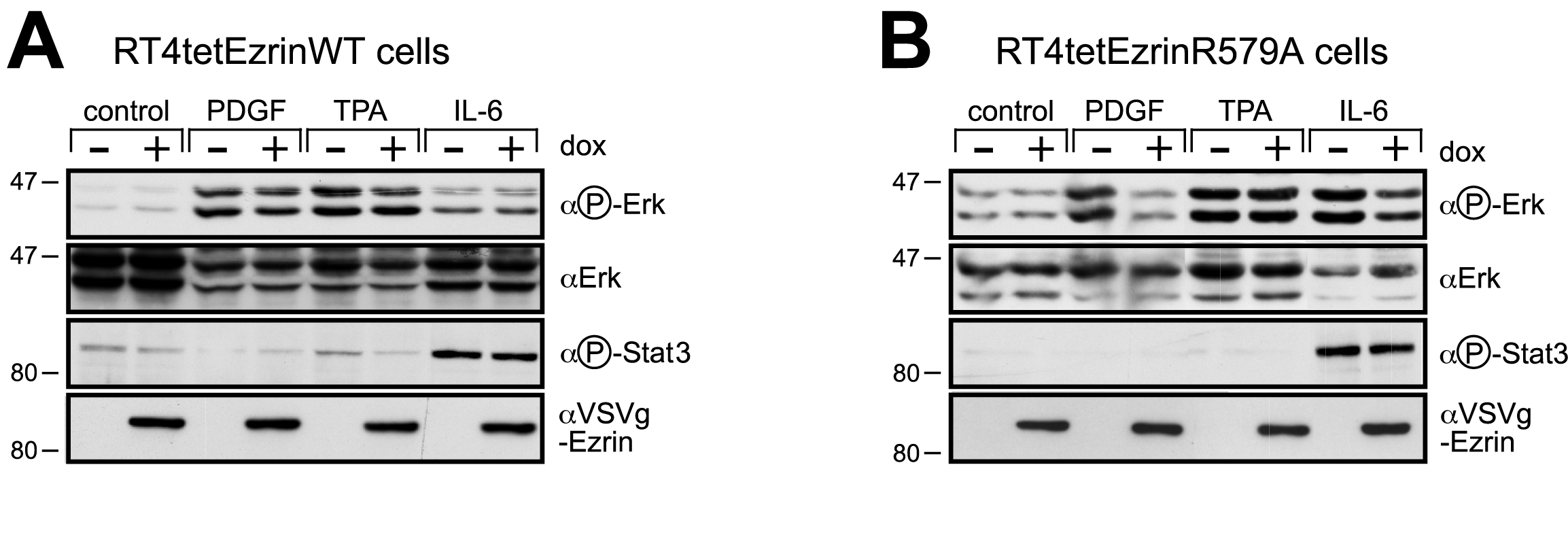

Supplement: Figure S2 — Ezrin R579A mutant inhibits PDGF-induced Erk phosphorylation. A, RT4 cells expressing dox-inducible ezrin wildtype-VSVg were plated at low density, serum starved overnight prior to treatment with either PDGF (20 µM, 5 min), TPA (100 ng/ml, 5 min) or IL-6 (1 ng/ml, 5 min). Lysates immunoblotted as indicated. B, RT4 cells expressing dox-inducible ezrin R579A mutant-VSVg were plated at low density, serum starved overnight prior to treatment with either PDGF (20 µM, 5 min), TPA (100 ng/ml, 5 min) or IL-6 (1 ng/ml, 5 min). Lysates immunoblotted as indicated. (TIF) [file pone.0027511.s002.tif]
